# A Method for Autonomous Navigation and Positioning of UAV Based on Electric Field Array Detection

**DOI:** 10.3390/s21041146

**Published:** 2021-02-06

**Authors:** Yincheng Li, Wenbin Zhang, Peng Li, Youhuan Ning, Chunguang Suo

**Affiliations:** 1 Faculty of Science, Kunming University of Science and Technology, Kunming 650504, China; liyincheng@stu.kust.edu.cn (Y.L.); suochunguang@kust.edu.cn (C.S.); 2 Faculty of Mechanical and Electrical Engineering, Kunming University of Science and Technology, Kunming 650504, China; lipeng@stu.kust.edu.cn (P.L.); ningyouhuan@stu.kust.edu.cn (Y.N.); 3 School of Astronautics, Harbin Institute of Technology, Harbin 150001, China

**Keywords:** overhead transmission line, UAVs, positioning and navigation, power line inspection, power frequency electric field sensor

## Abstract

At present, the method of using unmanned aerial vehicles (UAVs) with traditional navigation equipment for inspection of overhead transmission lines has the limitations of expensive sensors, difficult data processing, and vulnerable to weather and environmental factors, which cannot ensure the safety of UAV and power systems. Therefore, this paper establishes a mathematical model of spatial distribution of transmission lines to study the field strength distribution information around transmission lines. Based on this, research the navigation and positioning algorithm. The data collected by the positioning system are input into the mathematical model to complete the identification, positioning, and safety distance diagnosis of the field source. The detected data and processing results can provide reference for UAV obstacle avoidance navigation and safety warning. The experimental results show that the positioning effect of the positioning navigation algorithm is obvious, and the positioning error is within the range of use error and has good usability and application value.

## 1. Introduction

With the continuous expansion of overhead transmission lines and the rapid increase of coverage, the regional environment of overhead transmission lines is complex and diverse, which makes the operation and maintenance of overhead transmission lines more difficult. The traditional power transmission line inspection method has high strength, long cycle, high risk factors, and blind inspection area, which is difficult to meet the needs of modern power grid development [1,2]. Based on this, the unmanned aerial vehicle (UAV), as a scientific and efficient inspection method, has been widely concerned. Especially in recent years, a large number of academic and engineering applied research have been carried out to enable UAV to better complete advanced work such as search and rescue [3], path planning [4,5], target tracking [6,7], and so on. In order to achieve autonomous navigation and intelligent obstacle avoidance, a variety of guidance, navigation, and control algorithms have been developed, which has laid the foundation for the application research of drones in power patrol inspection. Furthermore, UAVs are widely used by domestic and foreign power companies for inspection and reconnaissance of transmission lines due to their advantages such as strong maneuverability, simple control, diverse forms, high efficiency, and low cost [8,9,10]. 

However, the current research based on UAV autonomous inspection of overhead transmission lines is still in its infancy. The main academic and engineering application research focuses on the detection of power transmission lines through UAVs equipped with lidar, visible light sensors, and infrared cameras, collecting fault data to provide navigation information for line patrol UAVs. The location and height of the data collected by the UAV depends on the vision of the flight control personnel and the detection results of these sensing devices. Therefore, the inspection of overhead transmission lines by UAVs depends heavily on the level of flight control of the control personnel, which is difficult to control manually and has low positioning accuracy, which affects aerial photography and status diagnosis [11,12,13]. To a large extent, this limits the operational performance of UAVs, and cannot make good use of the advantages of UAVs’ strong maneuverability, flexible control and high efficiency.

Therefore, UAV autonomous inspection of overhead high-voltage transmission lines has been studied, hoping that UAVs can perform more advanced and optimized aerial inspection tasks [14,15,16]. In these studies, the more feasible methods are the use of LiDAR technology and real-time kinematic (RTK) technology. Among them, LiDAR obtains high-precision 3D point cloud data of line channels to plan flight routes for autonomous inspection but extracting power line and tower information from disordered laser point clouds has a large amount of data processing. At this stage, it mainly depends on manual processing, the inspection efficiency is low [17,18,19,20,21,22]. Reference [17] uses a UAV equipped with lidar to detect the transmission line, extracts the characteristics of the transmission line through the acquired point cloud data, calculates the distance between the UAV and the transmission line, and diagnoses the safe distance to ensure UAV safety inspection. Reference [18] relies on artificially extracting features of ground objects from point cloud data and removing them, and then using the density features of the tower and transmission lines to locate the tower and transmission lines for navigation. Reference [19] made statistics on the detected point cloud data, extracted the characteristics of the target object, and mapped it to a two-dimensional plane, and detected the long direct power line based on Hough transform. Using laser radar detection technology for UAV autonomous patrol navigation, it is necessary to classify point cloud data, extract feature data, analyze and calculate the relationship between transmission wire and other ground objects, and then provide navigation information for UAV autonomous patrol [20,21,22]. RTK (real-time kinematic) technology can perform high-precision positioning for inspection UAVs, relying on RTK technology, the patrol UAV can perform patrol inspection according to the preset patrol inspection path, and achieve data collection at specific locations [23,24,25]. RTK technology has laid the foundation for automatic inspection of UAVs. In engineering applications, the State Grid Corporation of China currently detects line channels based on lidar technology, and China Southern Power Grid Corporation uses CW-LiDAR to measure the safe distance of the wire to ground. However, these are only at the level of simple application of equipment, and more advanced inspection techniques still need to be studied in depth.

As airborne lidar systems are expensive, complicated to operate, and bulky, many small electric power companies cannot afford expensive costs and cannot implement large-scale application promotion [26]. Extraction of power line features from image processing is a method with high feasibility, low cost, and easy to implement as navigation information for UAVs. Research on methods and technologies for extracting long and straight power lines from aerial images has been favored by scientific researchers and power companies [27,28,29,30]. These processing methods use visible light sensors to collect images or videos during the inspection process, based on the mathematical methods of image processing, the linear characteristics of long straight wires are extracted from the cluttered background. However, these methods have defects such as being susceptible to weather factors, large amount of data processing, low accuracy, and poor real-time performance. In order to realize autonomous navigation of inspection UAVs, some researchers also use GPS, ultrasonic sensors, ultraviolet, infrared, visible light sensors, and a combination of these sensors to conduct navigation research on inspection UAVs [31,32]. However, there are few studies on the field strength information of the space around the overhead transmission line as the navigation and obstacle avoidance of the UAV. Using the stable field strength around the transmission line as the information for the UAV’s obstacle avoidance navigation can effectively avoid the limitations of the traditional detection system with large volume and mass, which is not easy to carry on the drone. At the same time, it can also better solve the problems of the current inspection methods used, such as being greatly affected by environmental and weather factors, small wire diameter, difficult to detect, and difficult data processing.

In this paper, the field source positioning algorithm will be studied according to the electric field distribution rule around the transmission line, and the airborne power frequency electric field sensor and obstacle avoidance navigation system will be designed. Identify and locate the position of the field source from the detection and processing results of the field strength distribution information, and the diagnosis of the safety distance can realize the autonomous positioning navigation and safety warning during the UAV patrol inspection.

## 2. Materials and Methods

### 2.1. Analysis and Research of Navigation Information Mathematical Model

In order to accurately study the distribution rule of the electric field around the overhead transmission lines and provide accurate navigation information for the UAVs. It is necessary to model and analyze the transmission line and the field strength around the line according to the actual working conditions.

**Figure 1:**
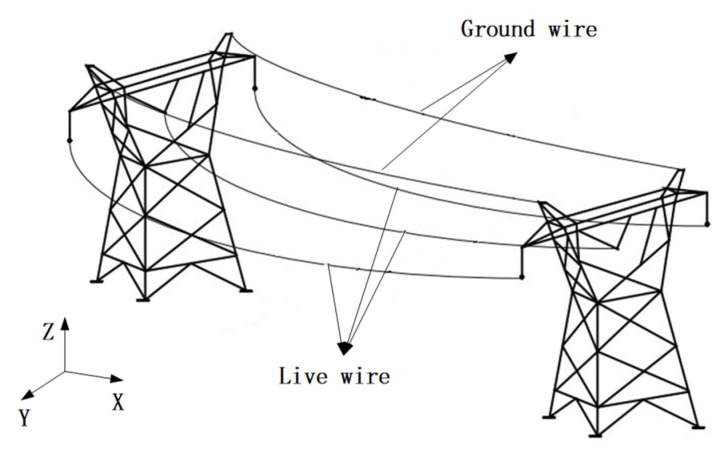
Three-dimensional model of high-voltage transmission line.

As shown in Figure 1, the power lines between the two power towers are distributed in a catenary shape due to their own gravity and external environmental factors. Assuming that the suspension point of the conductor is the origin of the coordinate system, the direction of the wire is in the X-direction, and the vertical direction is the Z-direction, then the catenary equation of the transmission line can be expressed as [33,34,35,36]:

(1) $$Z=\frac{\sigma_{0}h}{\gamma D_{h=0}}[\sinh\frac{\gamma d_{h}}{2\sigma_{0}}+\sinh\frac{\gamma (2x-d_{h})}{2\sigma_{0}}]-[\frac{2\sigma_{0}}{\gamma }\sinh\frac{\gamma x}{2\sigma_{0}}\sinh\frac{\gamma (d_{h}-x)}{2\sigma_{0}}]\sqrt{1+{\left(\frac{h}{D_{h=0}}\right)}^{2}}$$

where, *D_h_* = 0 can be expressed as:

(2) $$D_{h=0}=\frac{2\sigma_{0}}{\gamma }\sinh(\frac{\gamma d_{h}}{2\sigma_{0}})$$

where, *σ*_0_ is the stress at the lowest point of the overhang of the power line; *γ* is the ratio of the force of gravity per wire length to the cross section of the wire; and *d_h_* and *h* are the horizontal and vertical distances of the two suspension points of catenary, respectively.

It can be seen from the formula that the catenary equation contains a conic curve function, which is complicated to calculate and not easy to use. In practical engineering, oblique parabolic or flat parabolic formulas are often used to erect high-voltage transmission lines, so catenary formulas are generally simplified to oblique parabolic formulas or flat parabolic formulas [36]. The overhead transmission line studied in this subject has a small span, so a flat parabola is used instead of a catenary.

Assuming that the origin of the coordinates is the lowest point of the vertical line, the formula for flat parity is

(3) $$Z=\frac{\gamma x^{2}}{2\sigma_{0}}+h_{0}$$

where *h*_0_ is the height of the lowest point of the wire.

**Figure 2:**
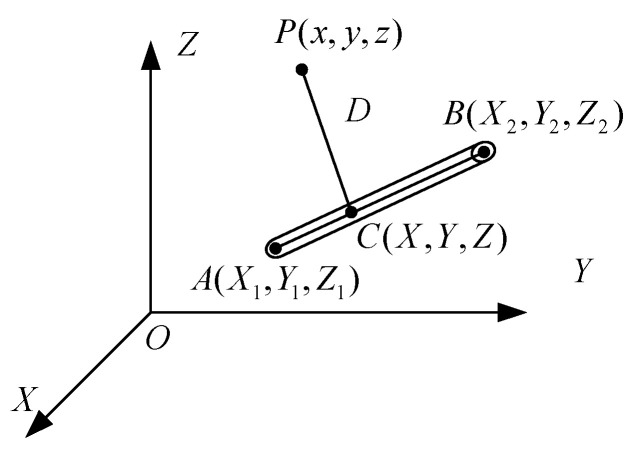
Field strength of a three-dimensional linear charge at a point in space.

The navigation and positioning system of UAVs accurately obtains navigation information, not only to establish an accurate spatial distribution model of wires, but to model and analyze the field strength information distributed around the wires. This study based on the idealized assumptions of the overhead transmission line model, according to the electrostatic field theory, comprehensive consideration of factors such as pitch, sag, and the layout of the conductors of each phase, the electric field generated by the high-voltage overhead transmission line in its surrounding space is analyzed. Assuming that a selected section of transmission line is used as the research object to analyze and calculate the field strength at a point in the surrounding space, the simulated charge type shown in Figure 2 is a linear charge unit with a linear distribution of charge density [37].

Assuming that the length of the line charge unit is *L* and *A* is the starting point, the coordinates of any point *C* (*X*, *Y*, *Z*) on the line unit can be expressed as Equation (4):

(4) $$\{\begin{array}{c} X(u)=X_{1}+\frac{l}{L}u\\ Y(u)=Y_{1}+\frac{m}{L}u\\ Z(u)=Z_{1}+\frac{n}{L}u \end{array}(0\leq u\leq 1)$$

Of which,

(5) $$\{\begin{array}{c} l=X_{2}-X_{1}\\ m=Y_{2}-Y_{1}\\ n=Z_{2}-Z_{1} \end{array}$$

the linear charge density at the beginning of the line unit is τ_1_, and the linear charge density at the end of the line unit is *τ*_2_, let *u = Lt* (0 ≤ *t* ≤ 1), the linear charge density at any point on the line charge can be expressed as

(6) $$\tau (u)=\tau_{1}+\frac{\tau_{2}-\tau_{1}}{L}u$$

Assuming the distance from the field source to the measurement point is *D*.

(7) $$D=\sqrt{{\left(X_{1}+lt-x\right)}^{2}+{\left(Y_{1}+mt-y\right)}^{2}+{\left(Z_{1}+nt-z\right)}^{2}}$$

Then the electric field strength generated by the line unit at any point P in the surrounding space is [38,39,40].

(8) $$\left\{ \begin{array}{l} E_{x}=\frac{L}{4\pi \varepsilon_{0}}\int_{0}^{1}\frac{\left(((\tau_{2}-\tau_{1})/L)t+\tau_{1})(x-X_{1}-lt\right)}{{\left(\sqrt{Rt^{2}+St+K}\right)}^{3}}dt\\ E_{y}=\frac{L}{4\pi \varepsilon_{0}}\int_{0}^{1}\frac{\left(((\tau_{2}-\tau_{1})/L)t+\tau_{1})(y-Y_{1}-mt\right)}{{\left(\sqrt{Rt^{2}+St+K}\right)}^{3}}dt\\ E_{z}=\frac{L}{4\pi \varepsilon_{0}}\int_{0}^{1}\frac{\left(((\tau_{2}-\tau_{1})/L)t+\tau_{1})(z-Z_{1}-nt\right)}{{\left(\sqrt{Rt^{2}+St+K}\right)}^{3}}dt \end{array}\right.$$

where

(9) $$\{\begin{array}{c} R=l^{2}+m^{2}+n^{2}\\ S=-2(l(x-X_{1})+m(y-Y_{1})+n(z-Z_{1}))\\ K={\left(x-X_{1}\right)}^{2}+{\left(y-Y_{1}\right)}^{2}+{\left(z-Z_{1}\right)}^{2} \end{array}$$

Assuming a certain range of distances, the conductors of each phase are parallel to each other, and they are brought into the flat parabolic Equation (3) of the transmission line. Based on the Equation (8), the superposition theorem is used to calculate the electric field strength around the transmission line to obtain the system of Equation (10):

(10) $$\left\{ \begin{array}{l} E_{x}=\frac{1}{4\pi \varepsilon_{0}}\sum_{n=1}^{N}\int_{x}\int_{0}^{1}\frac{\left(((\tau_{2}-\tau_{1})/L)t+\tau_{1})(x-X_{1}-lt\right)}{{\left(\sqrt{Rt^{2}+St+K}\right)}^{3}}\sqrt{1+4kx^{2}}dtdx\\ E_{y}=\frac{1}{4\pi \varepsilon_{0}}\sum_{n=1}^{N}\int_{x}\int_{0}^{1}\frac{\left(((\tau_{2}-\tau_{1})/L)t+\tau_{1})(y-Y_{1}-mt\right)}{{\left(\sqrt{Rt^{2}+St+K}\right)}^{3}}\sqrt{1+4kx^{2}}dtdx\\ E_{z}=\frac{1}{4\pi \varepsilon_{0}}\sum_{n=1}^{N}\int_{x}\int_{0}^{1}\frac{\left(((\tau_{2}-\tau_{1})/L)t+\tau_{1})(z-Z_{1}-nt\right)}{{\left(\sqrt{Rt^{2}+St+K}\right)}^{3}}\sqrt{1+4kx^{2}}dtdx \end{array}\right.$$

where *N* is the *N*-phase overhead transmission line. Then the electric field strength value of a point *P* in space can be expressed as

(11) $$E=\sqrt{E_{x}{}^{2}+E_{y}{}^{2}+E_{z}{}^{2}}$$

It can be seen from the analysis of Equations (10) and (11) that the field strength at a certain point in space has a definite relationship with the position of the field source. According to the mathematical model between the field strength of the measuring point and the spatial coordinates of the field source, using the UAV detection system as the origin of the coordinate system to establish a spatial rectangular coordinate system can locate the change of the position coordinate of the field source. The UAV can be navigated and located by locking changes in the location of the field source. However, it is difficult to locate the field source at a specific point on the overhead transmission line with multi-phase conductors under actual working conditions. To solve this problem, this paper uses the method of equivalent electric field strength amplitude to solve the problem of difficult positioning of multi-phase transmission conductors. Supposing there is a unit length of overhead transmission line at a point A (x, y, z) in space that produces a certain field strength *E* at the measurement point, at another point in space B (x_1_, y_1_, z_1_) there is also a hypothetical equivalent point charge that also produces the same electric field strength at the same measurement point. The space coordinate B (x_1_, y_1_, z_1_) of the equivalent charge can be calculated by the electric field sensor measuring the electric field intensity value. Then, the coordinates of A (x, y, z) are converted and solved through the spatial coordinate conversion relationship, so as to realize autonomous obstacle avoidance and navigation of the UAV.

It is assumed that the measurement point is the origin of the measurement coordinates, where the X axis is the forward direction during the UAV inspection, and the Z axis is the vertical direction. The field strength at the measurement point can be expressed as:

(12) $$\left\{ \begin{array}{l} E_{x}^{\prime }=\frac{Q_{0}{}^{\prime }}{4\pi \varepsilon_{0}}\frac{x^{\prime }}{D^{\prime }{}^{3}}\\ E_{y}^{\prime }=\frac{Q_{0}{}^{\prime }}{4\pi \varepsilon_{0}}\frac{y^{\prime }}{D^{\prime }{}^{3}}\\ E_{z}^{\prime }=\frac{Q_{0}{}^{\prime }}{4\pi \varepsilon_{0}}\frac{z^{\prime }}{D^{\prime }{}^{3}} \end{array}\right.$$

Of which,

(13) $$\{\begin{array}{c} D^{\prime }=\sqrt{x^{{}^{\prime }2}+y^{{}^{\prime }2}+z^{{}^{\prime }2}}\\ E^{\prime }=\sqrt{{E_{x}}^{{}^{\prime }2}+{E_{y}}^{{}^{\prime }2}+{E_{z}}^{{}^{\prime }2}} \end{array}$$

Therefore, the field strength value at the measurement point can be expressed as

(14) $$E^{\prime }=\frac{Q_{0}{}^{\prime }}{4\pi \varepsilon_{0}}\frac{1}{D^{\prime }{}^{2}}$$

The spatial position coordinates of the hypothetical equivalent charge *Q*′_0_ are calculated and solved by the sensor measurement value. Then, the spatial position coordinates of the line charges that generate the same electric field strength are calculated by the mathematical model, so as to locate the field source position of the transmission line and navigate the line patrol UAV. The field strength relationship generated by the two equivalent models in space should satisfy the relationship shown in Equation (15):

(15) $$\{\begin{array}{c} E_{x}^{\prime }=E_{x}\\ E_{y}^{\prime }=E_{y}\\ E_{z}^{\prime }=E_{z}\\ E^{\prime }=E \end{array}$$

### 2.2. Simulation Analysis of Electric Field Distribution Rule

Based on the above theoretical research, simulation software is used to simulate and analyze the effects of different voltage levels and different arrangement of transmission lines on the field strength distribution. The study found that with the same arrangement, the distribution of the electric field around transmission lines with different voltage levels is similar. The larger the voltage level, the higher the rate of change in electric field strength. The farther away from the transmission line, the smaller the rate of change in electric field strength, and the maximum field strength appears on the transmission line. 

**Figure 3:**
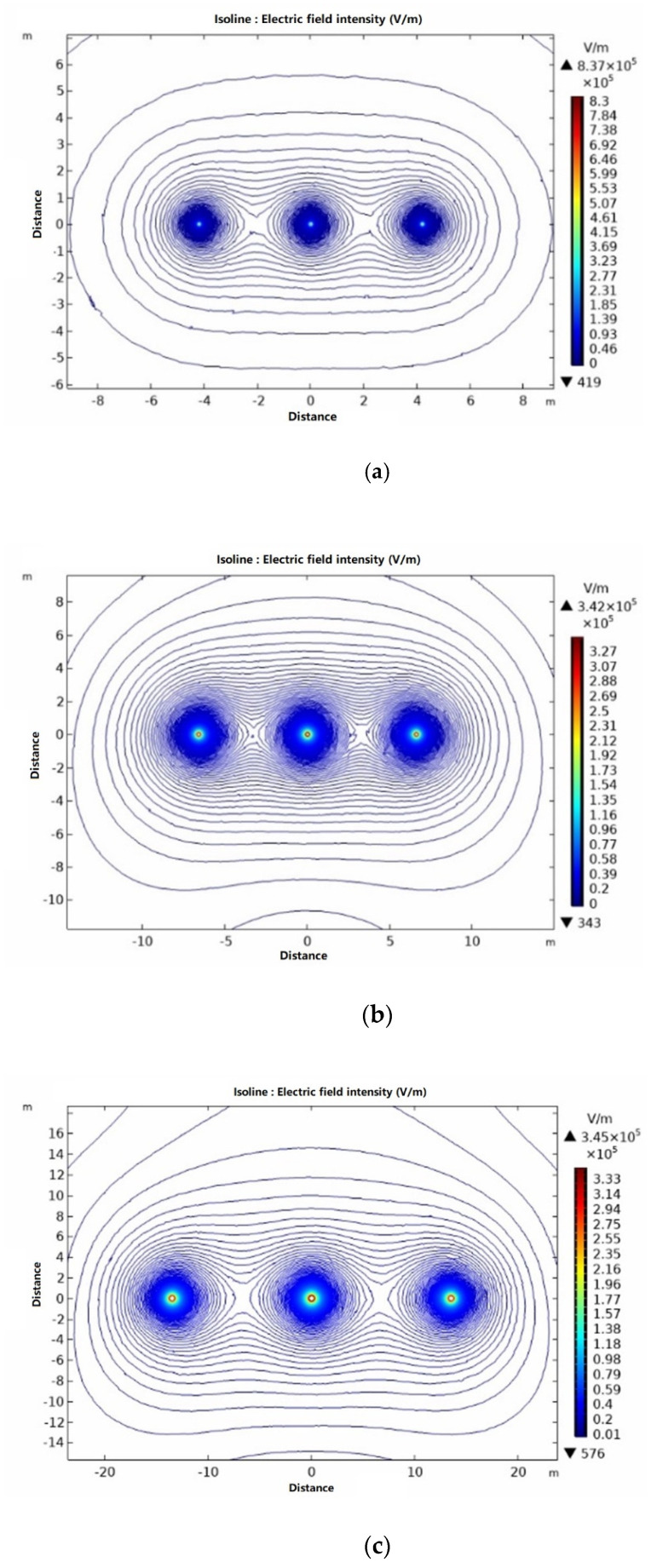
Field strength distribution curves of different voltage levels (**a**) Electric field intensity contour of 110 kV vertical section (**b**) Electric field intensity contour of 220 kV vertical section (**c**) Electric field intensity contour of 500 kV vertical section.

In this study, COMSOL simulation software is used to simulate and analyze the distribution law of electric field intensity around transmission lines with three voltage levels arranged horizontally. The analysis results are shown in Figure 3.

The analysis results show that for the same arrangement, the electric field distribution around the transmission lines with different voltage levels is roughly the same. The electric field strength decreases gradually away from the transmission line, and the maximum electric field strength is on the transmission line. Due to the superposition of the electric field vectors generated by each phase conductor, the electric field intensity below the edge phase conductor is strong, and the electric field intensity contour intersects between the two transmission lines. However, the change rates of electric field strength are different with different voltage levels. The larger the voltage level is, the higher the change rate of electric field strength is. At the same time, the electric field intensity of different voltage levels at the same position is not the same, therefore, the safety distance of UAV inspection increases with the increase of voltage level.

**Figure 4:**
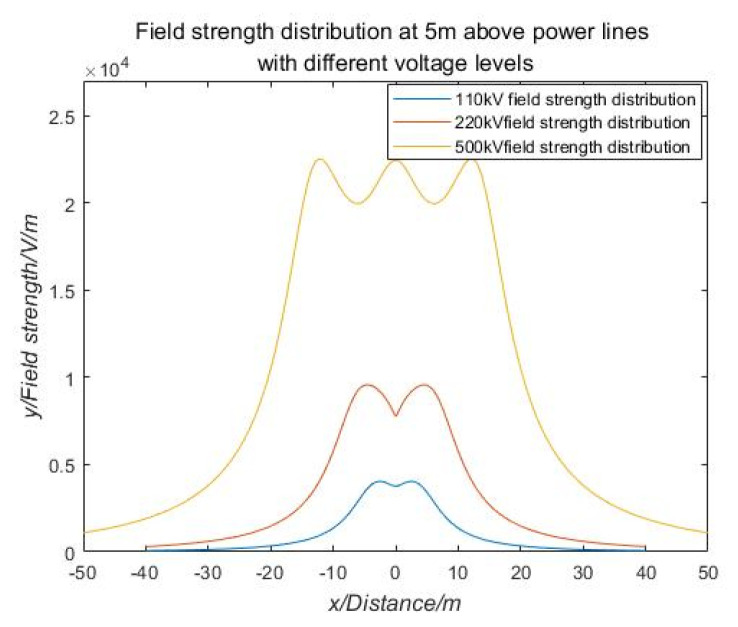
Distribution of field strength at 5 m above transmission lines of different voltage levels.

As shown in Figure 4, the field strength distribution of the 500 kV voltage level at 5 m above the transmission line is generally greater than the rate of change of the electric field strength of the two voltage levels of 220 kV and 110 kV.

**Figure 5:**
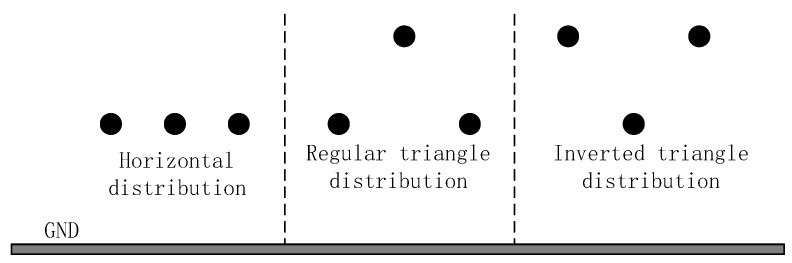
Distribution of overhead transmission lines.

**Figure 6:**
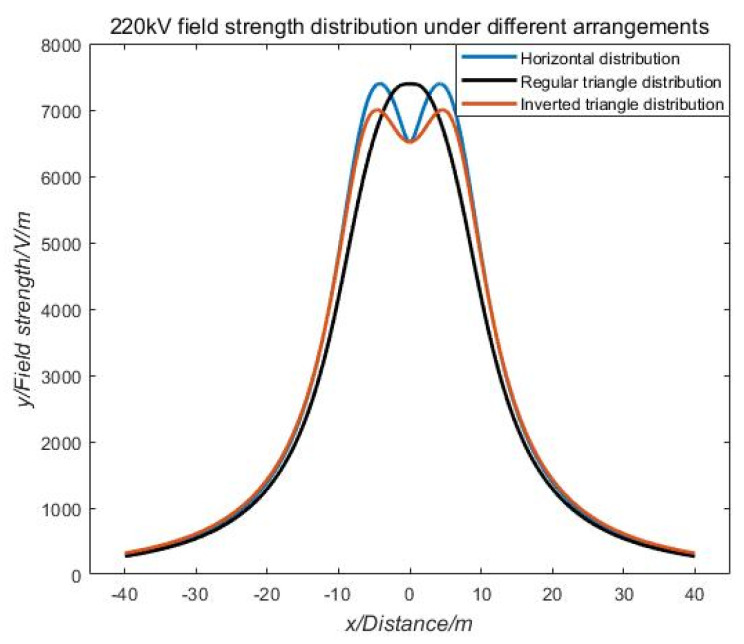
Electric field strength distribution in different arrangements.

As shown in Figure 5, there are different arrangements of common single-circuit transmission wires. According to the established mathematical model for the calculation of the electric field of the high-voltage transmission line, MATLAB simulation software was used to simulate and analyze the distribution of the electric field at a location 6 m above the transmission line with different arrangement of 220 kV voltage levels. The calculation results are shown in Figure 6.

As shown in Figure 6, the electric field distribution of horizontally and inverted triangle transmission lines is similar, showing an "M" shape, and the transmission field of a transmission line with a regular triangular arrangement has a unimodal electric field distribution. An extreme value of the electric field intensity distribution will always appear near the mid-phase conductor. By extracting this important feature, the patrol path of the UAV can be located.

**Figure 7:**
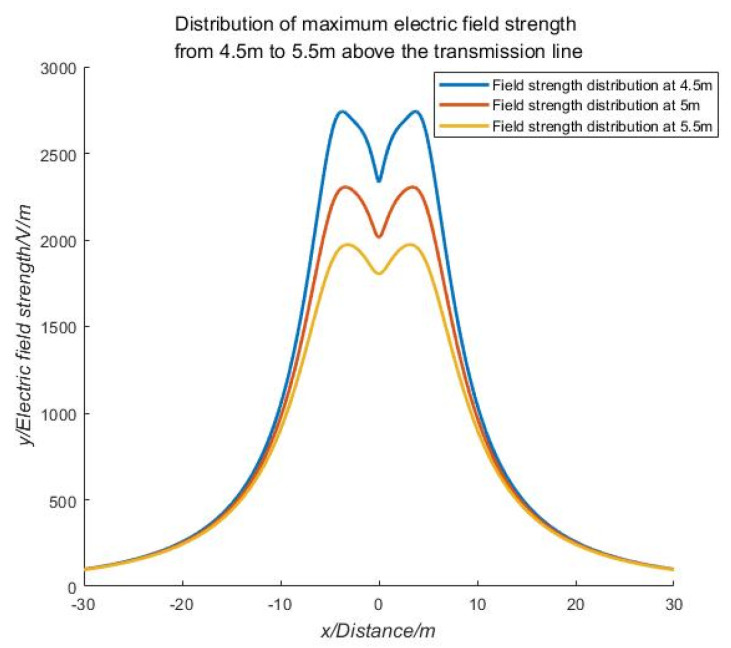
Electric field intensity distribution of 4.5 m–5.5 m above the transmission line.

**Figure 8:**
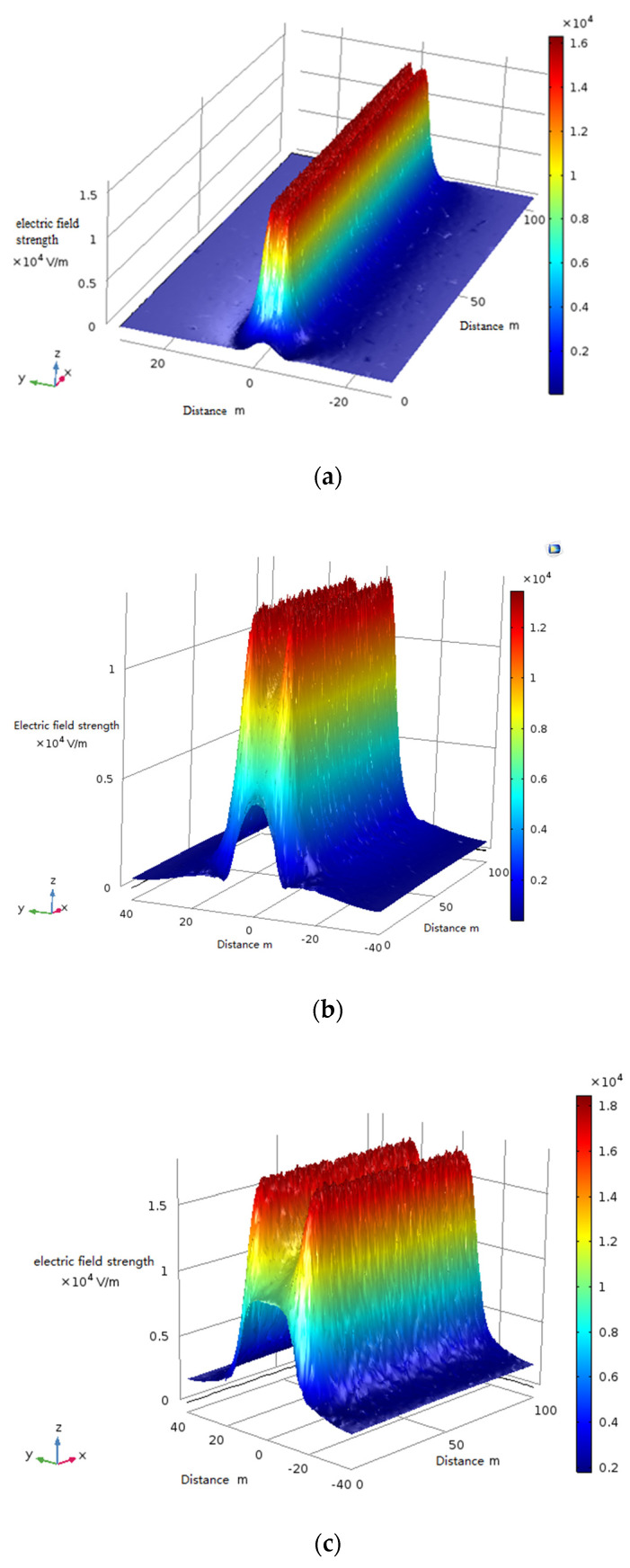
(**a**) Three-dimensional electric field intensity distribution map at 1.5 m above a 110 kV transmission line (**b**) Three-dimensional electric field intensity distribution map at 3 m above a 220 kV transmission line (**c**) Three-dimensional electric field intensity distribution map at 5 m above a 500 kV transmission line.

In practical engineering applications, the UAV’s trajectory cannot be completely maintained within the plane of the safe distance. The UAV may move up and down within the standard plane of the safety distance. Therefore, it is necessary to study the distribution law of electric field strength in the three-dimensional motion space of the UAV. Based on this, the field strength distribution law near the safety distance of 110 kV voltage level is selected for simulation analysis. The simulation analysis results are shown in Figure 7. The three-dimensional electric field intensity distribution above transmission lines with different voltage levels is shown in Figure 8.

According to the analysis in Figure 7 and Figure 8, it can be seen that the combined electric field strength curve along the X-axis direction along the top of the transmission line shows a hump shape in the same height range. Because the electric field vectors generated by the transmission conductors of different phases are superimposed on each other, an extreme value of the field strength distribution will always appear above or below the middle-phase conductor, and the closer to the conductor, the more obvious the distribution law and rate of change of the field strength. By detecting and identifying this abrupt point of field strength, the location of the transmission line as a field source can be achieved.

**Figure 9:**
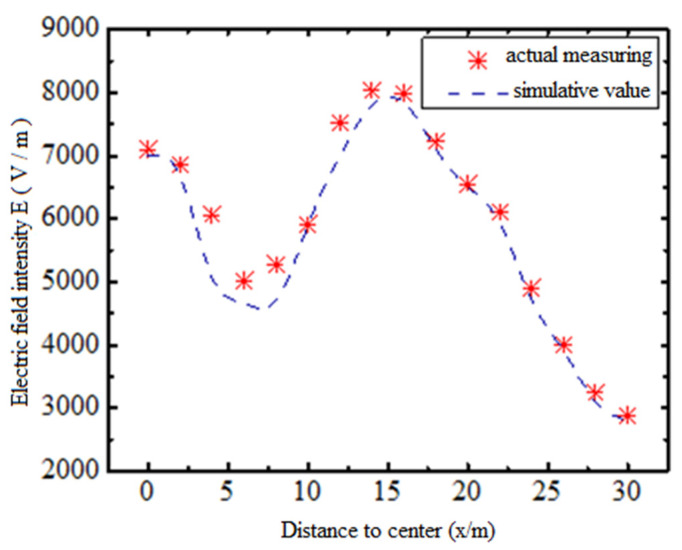
Comparison of measurement and simulation.

In order to verify the accuracy of the established simulation model and calculation method, the field strength signals collected under a certain actual working condition are compared with the established mathematical model. The collected data is the working condition of a relatively flat ground single-loop 500 kV overhead transmission line, and the measuring equipment is the low frequency electromagnetic field analyzer EHP-50 F and broadband measuring instrument NBM-550 of Narda company in Germany. The measuring equipment is installed in the position of 2 m away from the ground height, from the bottom of the transmission line lateral to the outside, the sampling point spacing in the measurement process is 2 m, the equipment is far from the obstacles such as the measuring personnel, reduce the influence on the electric field measurement, read the measurement data through the optical fiber communication interface, the measured results and the simulation results are shown in Figure 9.

It can be seen from Figure 9 that the measurement results have the same trend with the simulation results, and the maximum relative error is within 15%, which verifies the accuracy of the mathematical model analysis of field strength distribution and the rationality of the calculation method in this paper. The calculation error is within the allowable range of engineering application error.

### 2.3. Design and Research of Airborne Power Frequency Electric Field Sensor Probe

**Figure 10:**
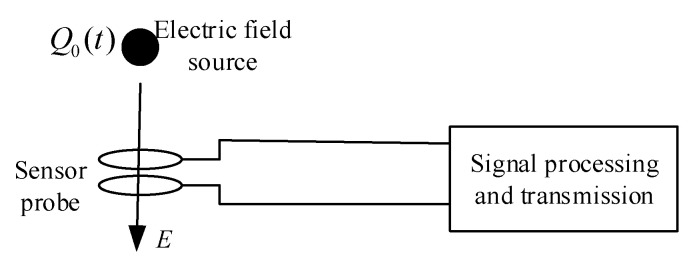
Schematic diagram of sensor measurement of electric field.

Collecting field strength distribution information around overhead transmission lines is used as a reference for UAV positioning and navigation and safety warning. It is necessary to design a portable power frequency electric field sensor suitable for UAVs for this application scenario. The schematic diagram is shown in Figure 10.

According to the principle of electrostatic induction, a conductor located in an alternating electric field has a surface induced charge that changes with time at the same frequency as the electric field to be measured. Based on this induced charge, a voltage or current signal proportional to the electric field to be measured can be obtained, thereby realizing the measurement of the electric field.

The surface density of the induced charge on the probe is σ, ε_0_ is the dielectric constant in the air, and the amount of induced charge generated by the change in the measured electric field strength *E*(*t*) is *Q*(*t*). This relationship can be expressed as [38,39].

(16) $$Q(t)=\int \sigma ds=\varepsilon_{0}E(t)S$$

where *S* is the effective area of the induction plate.

**Figure 11:**
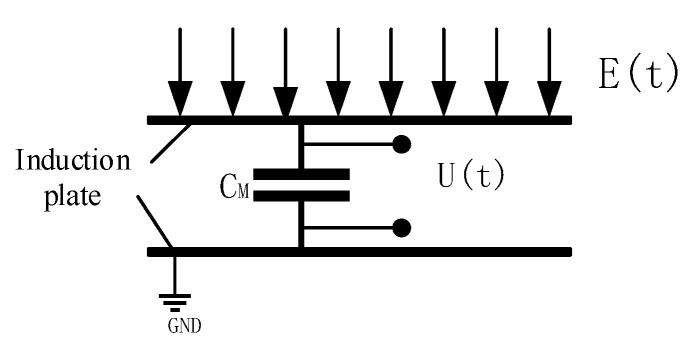
Schematic diagram of sensor probe structure.

**Figure 12:**
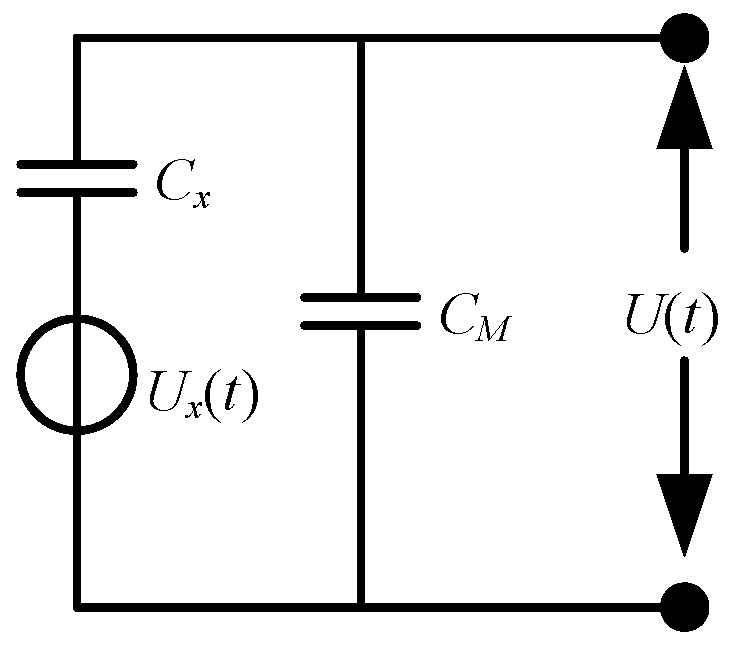
Induction unit equivalent circuit diagram.

**Figure 13:**
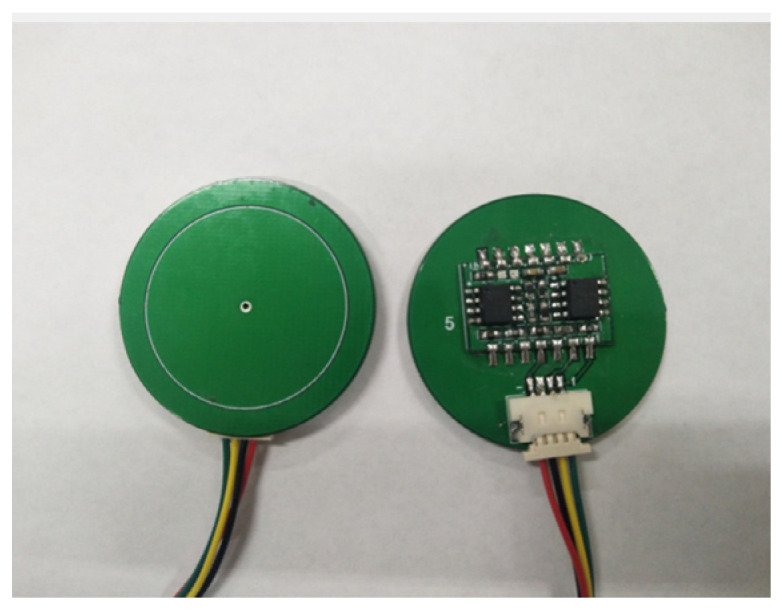
Airborne power frequency electric field sensor probe.

The structure of the electric field induction unit designed according to this principle is shown in Figure 11, which is composed of three parts: an upper plate, a lower plate, and a sampling capacitor. The equivalent circuit diagram is shown in Figure 12. The physical diagram of the designed airborne power frequency electric field sensor is shown in Figure 13.

According to the analysis of the equivalent circuit schematic diagram 10, The voltage signal *U*_(*t*)_ formed at the two ends of the sampling capacitor *C_M_* by the induced charge generated on the sensing plate is used as the output signal, and their relationship can be expressed as

(17) $$U(t)=\frac{kU_{x}\left(t\right)C_{x}}{C_{x}+C_{s}}=\frac{kQ(t)}{C_{x}+C_{s}}$$

where *C_x_* is the inherent capacitance of the sensing unit, and *k* is the correction factor related to the structure of the sensing unit.

From the analysis of Equations (14), (16), and (17), we can know that through the processing and calculation of the output voltage signal of the sensing unit, the electric field strength information and the induced charge of the detection electrode plate can be obtained.

## 3. Research on Positioning and Navigation Algorithms

In this paper, according to the distribution rule of electric field intensity, the field source positioning model is established by the method of signal array detection, and the spatial rectangular coordinate system is established with the measurement point as the origin. The location of the field source is P (x_0_, y_0_, z_0_), according to the analysis of the measurement model of the sensor, it can be known that when the radius of the probe of the round sensor is much smaller than the distance between the sensor and the measurement point, the voltage signal output by the sensor has a relationship as shown in Equation (18) with the amount of charge carried by the field source.

(18) U(t)(Q0(t),x,y,z)=P⋅z(x2+y2+z2)32⋅Q0(t)⋅S

where *U*_(*t*)_ is the voltage signal output by the sensor, *Q*_0_(*t*) is the amount of charge carried by the electric field source, and *P* is the proportional constant related to the sensor parameters.

From the analysis of Equation (18), it can be seen that the output voltage signal of the sensor contains the spatial position information of the field source and the charge information of the field source. Therefore, only four equations need to be established to solve the four unknown parameters, and then to locate the relative position of the field source. According to the analysis, only four identical electric field sensors need to be installed in a specific layout relationship to form a measurement system to complete the solution of the field source spatial position. The measurement relationship of each sensor satisfies the Equation (18), and the simultaneous solution can obtain the Equation (19).

(19) $$\{\begin{array}{c} U_{1}=PQ_{0}S\cdot \frac{z_{0}}{D_{1}^{3}}\\ U_{2}=PQ_{0}S\cdot \frac{z_{0}}{D_{2}^{3}}\\ U_{3}=PQ_{0}S\cdot \frac{z_{0}}{D_{3}^{3}}\\ U_{4}=PQ_{0}S\cdot \frac{z_{0}}{D_{4}^{3}} \end{array}$$

where *D_i_* (*i* = 1, 2, 3, 4) is the distance between the *i*-th sensor and the field source.

**Figure 14:**
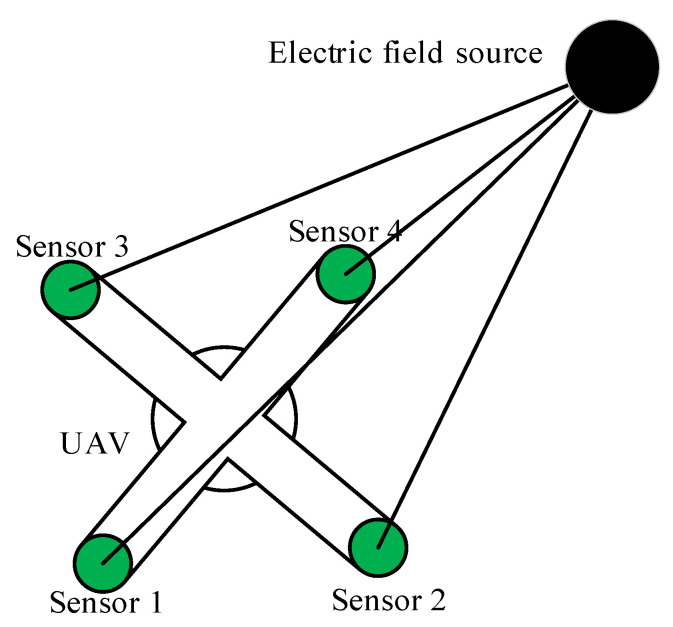
Sensor detection plate array layout [41].

The structure model of the constructed measurement system is shown in Figure 14 [41], where the position coordinates of the four sensor probes are (0,0,0), (L,0,0), (0,L,0), (L,L,0), each detection plate is a circle of area S, then the distance *D_i_* (*i* = 1, 2, 3, 4) between the field source and each detection plate can be expressed as

(20) $$D_{1}=(x_{0}^{2}+y_{0}^{2}+z_{0}^{2}{)}^{\frac{1}{2}}$$

(21) $$D_{2}=({\left(x_{0}-L\right)}^{2}+y_{0}^{2}+z_{0}^{2}{)}^{\frac{1}{2}}$$

(22) $$D_{3}=(x_{0}^{2}+{\left(y_{0}-L\right)}^{2}+z_{0}^{2}{)}^{\frac{1}{2}}$$

(23) $$D_{4}=({\left(x_{0}-L\right)}^{2}+{\left(y_{0}-L\right)}^{2}+z_{0}^{2}{)}^{\frac{1}{2}}$$

Simultaneous Equations (19)–(23) can solve the position coordinates of the field source:

(24) $${\left(\frac{(\frac{U_{1}}{U_{4}}{)}^{\frac{2}{3}}+1+\frac{2}{L}}{2+2\cdot L}-\frac{\frac{1}{L}[((\frac{U_{1}}{U_{2}}{)}^{\frac{2}{3}}+(\frac{U_{1}}{U_{3}}{)}^{\frac{2}{3}})\cdot (1+L)]}{2+2\cdot L}\right)}^{-\frac{1}{2}}{}^{}$$

(25) $$D_{2}=(\frac{U_{1}}{U_{2}}{)}^{\frac{1}{3}}\cdot D_{1}$$

(26) $$D_{3}=(\frac{U_{1}}{U_{3}}{)}^{\frac{1}{3}}\cdot D_{1}$$

(27) $$x_{0}=\frac{\left(L^{2}+D_{1}^{2}-D_{2}^{2}\right)}{2L}$$

(28) $$y_{0}=\frac{\left(L^{2}+D_{1}^{2}-D_{3}^{2}\right)}{2L}$$

(29) $$\begin{array}{cc} z_{0} & =[(-2L^{4}+2L^{2}\cdot D_{2}^{2}-2D_{1}^{4}+2D_{1}^{2}D_{2}^{2}\\ & -D_{2}^{4}+2L^{2}\cdot D_{3}^{2}-D_{3}^{4}{)}^{\frac{1}{2}}]/2L \end{array}$$

It can be seen from the above equations that the field source position coordinates (*x*_0_, *y*_0_, *z*_0_) can be solved by the output voltage signal U of the sensor and the distance L between the sensors. Therefore, the accurate positioning of the relative position of the electric field source and the drone can be achieved, which can provide a reference for the UAV obstacle avoidance navigation and safety warning.

## 4. Design of Power Line Positioning and Navigation System for UAV Inspection

**Figure 15:**
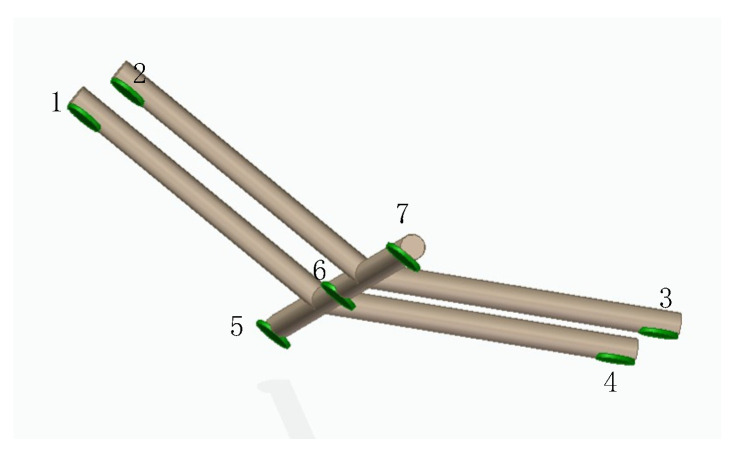
Electric field information positioning system for inspection drone.

Based on the study of the distribution law of field strength around overhead transmission lines, analysis of Figure 7 and Figure 8 shows that the distribution law of field strength above the transmission line is hump-shaped. The characteristic signals of peaks or troughs can be extracted from the field strength detection data, and the patrol path of the patrol drone can be locked near the peaks or troughs of the electric field distribution. In other words, the detection information of the field strength distribution regulation can provide a reference basis for the UAV navigation. Therefore, the designed positioning and navigation system can not only detect the mutation information of the electric field around the overhead transmission line, but also locate the field source according to the detected electric field information. Based on the above analysis of the mathematical model of the navigation and positioning system, an obstacle avoidance navigation system as shown in Figure 15 is established.

The numbered discs in Figure 15 represent power frequency electric field sensors. Each sensor is installed in a specific geometric relationship through an intermediate bracket. 3, 4, 5, 6, and 7 are located on a plane for horizontal navigation. The analysis of the established mathematical model of navigation shows that the five sensors can be arranged in a linear array to detect and identify sudden changes in the field intensity distribution such as peaks or troughs, thereby achieving navigation of the drone, and guide the inspection drone to conduct inspections along the transmission lines above the overhead transmission lines. Install 1, 2, 3, and 4 sensors in a specific geometric relationship in the same plane, by real-time detection of the field strength around the overhead transmission line and using the detected field strength information as the input of the positioning model, the positioning and ranging of the field source can be completed.

## 5. Experiment and Result Analysis

### 5.1. Verification of Positioning Algorithm in Laboratory Environment

**Figure 16:**
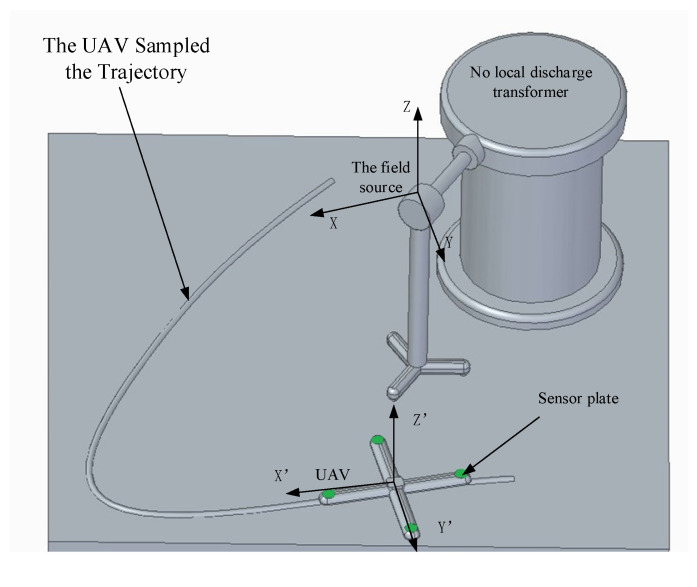
Schematic diagram of the experimental data acquisition system [41].

In order to verify the practicability and accuracy of the positioning and navigation algorithm proposed in this paper, the laboratory non-partial power frequency high-voltage experimental platform is used as the electric field source to verify the feasibility of the field source positioning algorithm in the laboratory environment. Based on the engineering background of the UAV patrol transmission line, the model of the four-rotor UAV selected in the experiment is DJI-NAZA, the size is 0.68 m × 0.68 m × 0.4 m, the material is carbon fiber, and the hover stability is good. The radius of the designed circular sensor probe is 0.022 m, and these sensors are respectively mounted on the four wings of the UAV in a specific positional relationship. The schematic diagram of the relevant experimental layout structure is shown in Figure 16.

**Figure 17:**
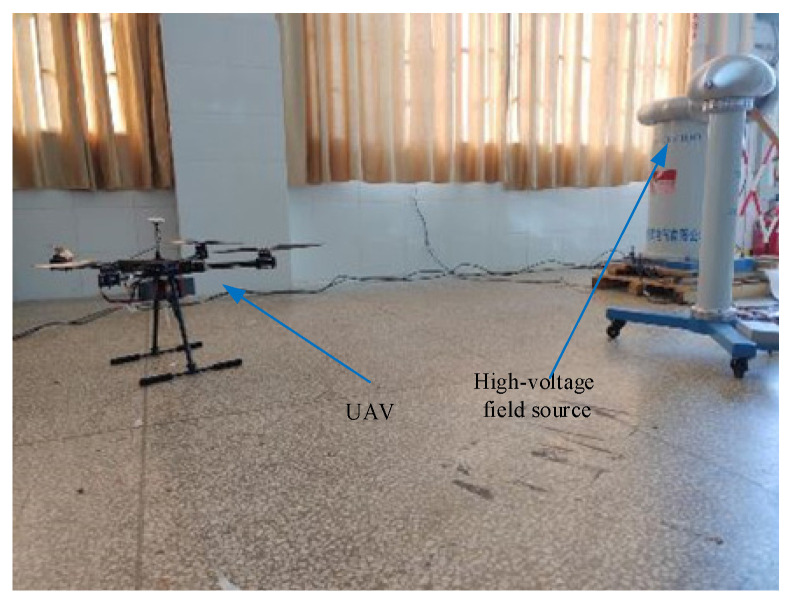
Lab test scene layout.

During the experiment, a circular sampling trajectory with a radius of 2 m was first arranged with the field source as the origin of the coordinate system, and 10 sampling points were taken at equal intervals on the trajectory. Then adjust the output voltage of the transformer to 10 kV in order to generate a power frequency electric field of suitable size to the space. Finally, the UAV equipped with the sensor measurement system is slowly moved to the sampling point and hovered, and the output voltage signal value of each sensor and the position coordinates of the sampling point are recorded. After the data collection is completed, move to the next sampling point for signal collection until the experimental data collection at the last sampling point is completed. The experimental scene graph is shown in Figure 17.

The sensor system mounted on the UAV hovered at 10 sampling points to collect electric field information, and record the value of the output voltage signal of each sensor at each sampling point, use the recorded voltage signal value as the input of the positioning mathematical model, and calculate the position coordinate of the output field source after calculation and solution. In this experiment, due to the small height difference between the UAV and the field source, the z-coordinate obtained by the solution is basically unchanged, and it can be ignored. The mathematical analysis software MATLAB is used to fit and analyze the (x, y) coordinate points. Remove a distortion point and fit the remaining nine coordinate points as shown in Figure 16.

It can be known from the analysis of the fitting curve shown in Figure 16 that the fitting curve of the coordinate points output by the positioning mathematical model can well restore the true trajectory of the UAV movement. This proves the scientific rationality of the positioning mathematical model and the feasibility of engineering application. The analysis results of the positioning error show that the positioning effect of the positioning algorithm is obvious, and the positioning error is within the range of the actual application error, which can meet the UAV navigation and positioning requirements of the actual line inspection conditions.

### 5.2. Error Analysis

The relative error is used to quantify and analyze the credibility of the measured value. The value obtained by using the percentage of the absolute error caused by the measurement to the measured true value represents the relative error [41]. Assuming the measurement value is $\bar{x}$ and the measured true value is *u*, the relative error *δ* can be expressed as

(30) $$\delta =\frac{\;\bar{x}\;-u}{u}\times 100\%$$

**Figure 18:**
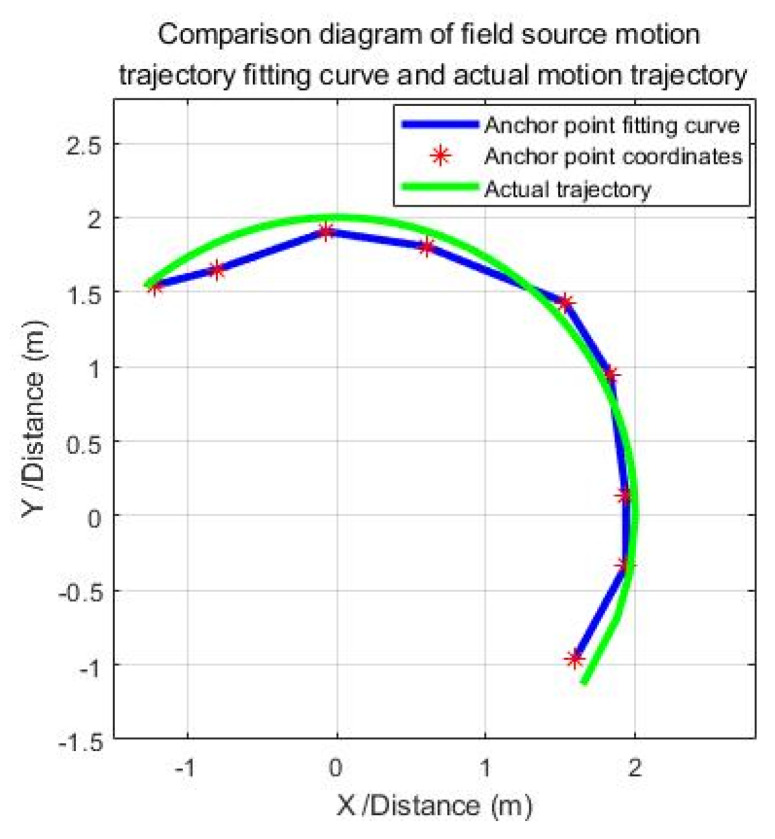
Field source motion trajectory coordinate fitting curve.

**Figure 19:**
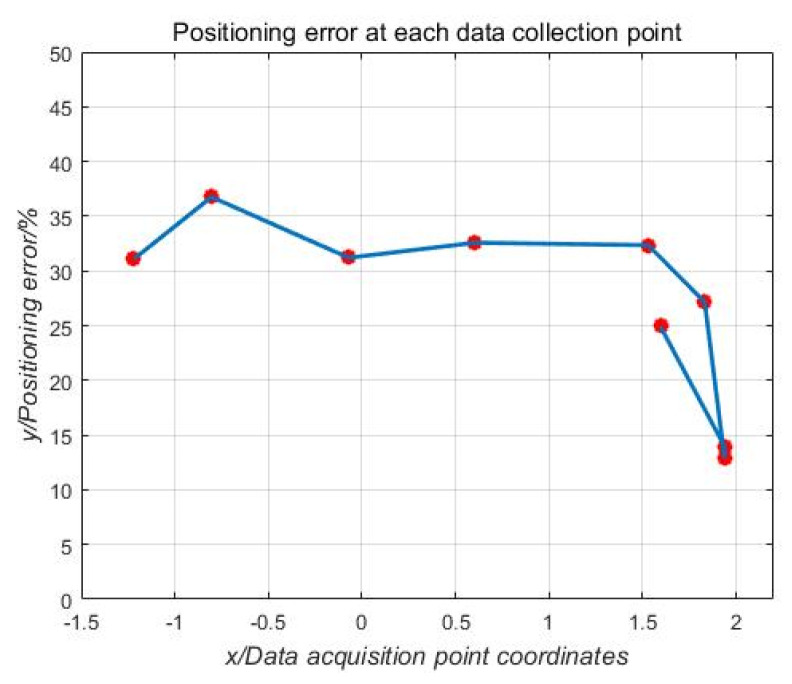
Positioning error curve for each anchor point.

The error curve obtained by performing error analysis on the fitting curve shown in Figure 18 is shown in Figure 19.

From the analysis in Figure 19, it can be seen that the positioning error in this experiment is about 30%, but most of this error is caused by the rough sensor and rounding errors during data processing. For example: obstacles such as drones in the electric field will have a greater impact on the distortion of the electric field, resulting in measurement errors on the sensors it carries; because the sensor measurement data is weak during the experiment, a small rounding error in data processing will have a greater impact on the results; the proximity of each sensor when installed and the low accuracy of the sensor consistency result in the measured data of the sensor not fully achieving the ideal positioning accuracy of the mathematical model; and due to the low height during the processing of the experimental data, the height was rounded down, resulting in calculation errors.

Therefore, in order to reduce the positioning error, the sensor should be installed as far as possible to avoid the position that interferes with the electric field or the sharp part of the carrier. At the same time, each sensor should be installed on the same horizontal plane, and the distance between each sensor should be as large as possible, so that the measured value gap between the two sensors is large, which is helpful to reduce positioning errors.

### 5.3. Experimental Results and Analysis

**Figure 20:**
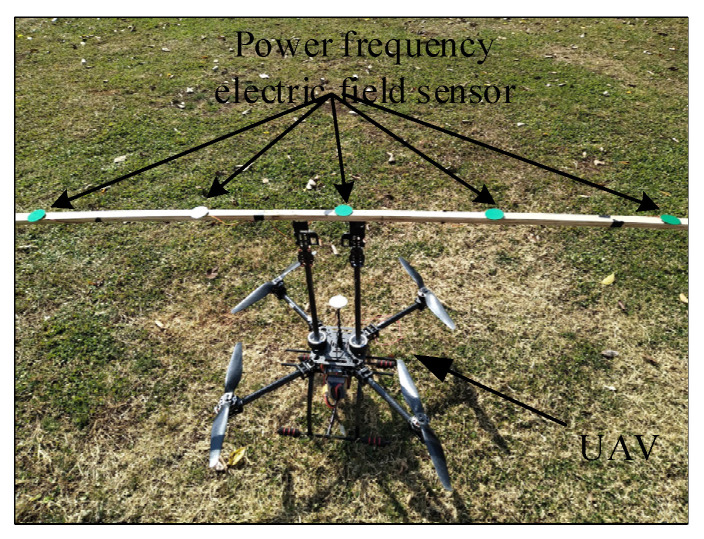
Inspection of unmanned aerial vehicle (UAV) with electric field measurement and navigation system.

**Figure 21:**
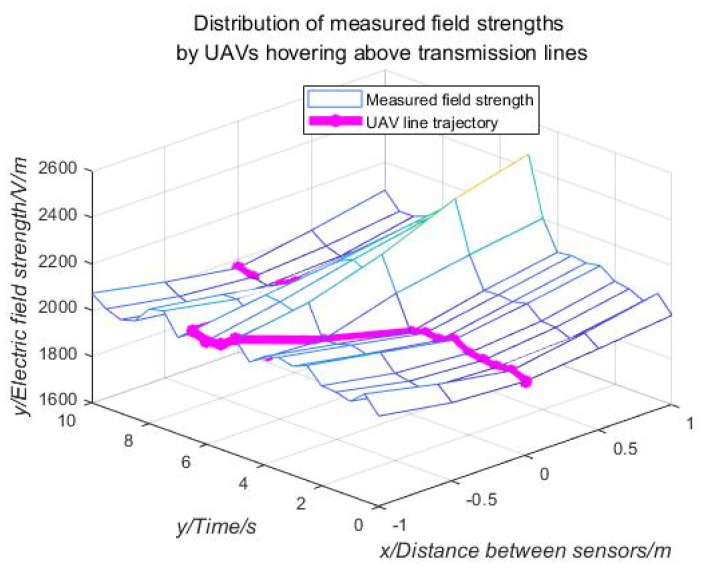
Electric field strength distribution measured by a drone hovering over a transmission line.

In order to verify the reliability of the autonomous navigation system and the scientificity of the navigation algorithm, the electric field measurement and navigation system was mounted on a quadrotor drone to conduct an inspection of the actual 110 kV transmission line. The physical picture of the patrol UAV equipped with the obstacle avoidance navigation system based on electric field measurement is shown in Figure 20. In order to reduce the error caused by the drone and environmental factors, select a clear and windless environment for flight testing. Control the UAV to fly above the medium-phase wire to hover to collect field strength data, select the data collected during the 10 s time period for analysis, and collect a data point for each sensor every 0.5s. The mathematical analysis software MATLAB was used to fit the collected experimental data. The fitted curve is shown in Figure 21.

As shown in Figure 21, the curve marked with a purple line is the trajectory of the UAV detected and recognized by the navigation system. It can be clearly seen that the experimental drone hovering over the transmission line is stable in the time period of 0–3.5 s; however, in the 3.5–7 s time period, the UAV drifted to the right of the direction of movement in a small range; Due to the robustness of the drone control, the drone drifted to the left in the forward direction during the 7–9 s period, and finally moved to hover near the square of the transmission line.

From the analysis of the collected data, it can be seen that the UAV is equipped with five power frequency electric field sensors to detect the electric field distribution of overhead transmission lines. According to the analysis and research of the electric field distribution law, it is found that the extreme value of the electric field intensity always appears above or below the medium-phase transmission line. It is possible to detect the field strength around the transmission conductor by using the sensor array layout, extract the information of the mid-phase conductor by processing the data, and then lock the trajectory of the UAV near the mid-phase conductor. Therefore, the collection and processing of the field strength distribution information around the overhead transmission line can provide a reference for UAV navigation and obstacle avoidance.

## 6. Summary

In this paper, based on the establishment of a mathematical model of the spatial distribution of overhead transmission lines, the field strength distribution law around the transmission lines is modeled and analyzed. Based on the specific distribution information of the electric field strength, a navigation and positioning mathematical model of the patrol UAV is established. Then on this basis, a navigation and positioning system is designed. During the UAV inspection process, the navigation and positioning system is equipped to detect the field strength distribution information in real time, and the collected field strength data is used as the input of the navigation and positioning mathematical model to identify and locate the position of the field source relative to the drone. The diagnosis of the field source safety distance provides reference basis for patrol UAV obstacle avoidance navigation and safety warning. The navigation warning method proposed in this paper has the advantages of less influenced by environment and weather, simple data processing, strong real-time performance, and lower cost. Finally, the sensor navigation system designed by the UAV is used to measure the electric field strength around the overhead transmission line in real time, the field source positioning algorithm is tested. The analysis of the experimental results verifies the reliability and rationality of the mathematical model.

## Data Availability

Data was obtained from [baidu netdisk] and are available [from the authors] with the permission of [baidu netdisk].

## References

[1] Chen X. (2019). Application and Development Prospect of Drones in Transmission line Inspection. Electr. Power Syst. Equip..

[2] Zhang X.Q., Su J.J. (2016). UAV Inspection Technology for Overhead Transmission Lines.

[3] Tomic T., Schmid K., Lutz P., Domel A., Kassecker M., Mair E., Grixa I.L., Ruess F., Suppa M., Burschka D. (2012). Toward a Fully Autonomous UAV: Research Platform for Indoor and Outdoor Urban Search and Rescue. IEEE Robot. Autom. Mag..

[4] Yao P., Xie Z., Ren P. (2017). Optimal UAV Route Planning for Coverage Search of Stationary Target in River. IEEE Trans. Control. Syst. Technol..

[5] Zhao Y., Zheng Z., Liu Y. (2018). Survey on computational-intelligence-based UAV path planning. Knowledge-Based Syst..

[6] Zhu S., Wang D., Low C.B. (2012). Ground Target Tracking Using UAV with Input Constraints. J. Intell. Robot. Syst..

[7] Yao P., Wang H., Su Z. (2015). Real-time path planning of unmanned aerial vehicle for target tracking and obstacle avoidance in complex dynamic environment. Aerosp. Sci. Technol..

[8] Zhang F., Wang W., Zhao Y., Li P., Lin Q., Jiang L. Automatic diagnosis system of transmission line abnormalities and defects based on UAV. Proceedings of the 2016 4th International Conference on Applied Robotics for the Power Industry (CARPI).

[9] Chen J.G., Yao P., Yang J.W. (2019). Application Research of Unmanned aerial Vehicle in Overhead Transmission Line Inspection. Hunan Electr. Power.

[10] Zhou G., Yuan J., Yen I.L., Bastani F. Robust real-time UAV based power line detection and tracking. In Proceeding of the 2016 IEEE International Conference on Image Processing (ICIP).

[11] Peng X.Y., Chi C., Rao Z.Q., Yang B.S., Mai X.M., Wang K. (2015). Safety Inspection and Intelligent Diagnosis of Transmission Line Based on Unmanned Helicopter of Multi Seneor Data Acquisition. High Volt. Eng..

[12] Zeng Y.H., He T., Guo S., Xiong Y.L., Cui Y.R. (2019). Research on Multi-rotor UAV Intelligent Power Line Inspection Based on Differential Positioning. Electr. Power.

[13] Cao F.H., Song Z.W., Li Y., Liu X., Qiao Y.J., Xin X.Q. (2016). Determination of precise spatial position of drone aerial photography. Bull. Surv. Map.

[14] Xiong D. (2014). The Research and Application of Path Planning for UAV Inspection Transmission Line. Master’s Thesis.

[15] Shao G.W., Liu Z., Fu J., Tan J.Y., Chen Y., Zhou L.W. (2020). Research Progress in Unmanned Aerial Vehicle Inspection Technology on Overhead Transmission Lines. High Volt. Eng..

[16] Peng X.Y., Song S., Qian J.J. (2017). Research on Automatic Positioning Algorithm of Power Transmission Towers Based on UAV LiDAR. Power Syst. Technol..

[17] Chen C., Mai X.M., Song S., Peng X.Y., Xu W.X., Wang K. (2015). Automatic Power Lines Extraction Method from Airborne LiDAR Point Cloud. Geomat. Inf. Sci. Wuhan Univ..

[18] You A.Q., Han X.Y., Li S.P., Yan Z.J. (2013). Transmission Lines Fitting and Towers Positionging in LiDAR Point Cloud. Comput. Sci..

[19] Li Z., Walker R., Hayward R. Advances in vegetation management for power line corridor monitoring using aerial remote sensing techniques. Proceedings of the (CARPI) 2010 1st International Conference on Applied Robotics for the Power Industry.

[20] Qin X., Wu G., Lei J., Fan F., Ye X. (2018). Detecting Inspection Objects of Power Line from Cable Inspection Robot LiDAR Data. Sensors.

[21] Qin X., Wu G., Ye X., Huang L., Lei J. (2017). A Novel Method to Reconstruct Overhead High-Voltage Power Lines Using Cable Inspection Robot LiDAR Data. Remote. Sens..

[22] Matikainen L., Lehtomäki M., Ahokas E., Hyyppä J., Karjalainen M., Jaakkola A., Kukko A., Heinonen T. (2016). Remote sensing methods for power line corridor surveys. ISPRS J. Photogramm. Remote. Sens..

[23] Peng X.F., Lv R., Li L.G., Yu W.M. (2019). RTK Technology Research in Navigation and Positioning. Geomat. Spat. Inf. Technol..

[24] Liu J.H., Tu R., Zhang P.F., Zhang R., Lu X.C. (2019). The model and accuracy analysis of combined RTK by GPS, GLONASS and BDS. Sci. Surv. Mapp..

[25] Wan H., He Y. (2016). Study on Application of UAV Photogrammetry with RTK in Water Transport Engineering. Beijing Surv. Mapp..

[26] Gao Z.G. (2010). The Research of Terrestrial Laser Scanning Data Processing and Modeling. Master’s Thesis.

[27] Yang L., Li H.F., Jiang W.S. (2019). Automatic Extraction of Power Lines Based on Gradient Symmetry from UAV Image. Geomat. World.

[28] Von Gioi R.G., Jakubowicz J., Morel J.-M. (2012). LSD: A lin segment detector. IPOL J..

[29] Sharma H., Bhujade R., Adithya V., Balamuralidhar P. Vision-based detection of power distribution lines in complex remote surroundings. Proceedings of the 2014 Twentieth National Conference on Communications (NCC).

[30] Cerón A., Mondragón I.F., Prieto F. (2014). Towards Visual Based Navigation with Power Line Detection. Advances in Visual Computing, Proceedings of the International Symposium on Visual Computing, Las Vegas, NV, USA, 8–10 December 2014.

[31] Zhang W., Ning Y., Suo C. (2019). A Method Based on Multi-Sensor Data Fusion for UAV Safety Distance Diagnosis. Electronics.

[32] Zheng T.R., Sun L.M., Lou T.T., Guo X., Liu Q.H. (2018). Determination Method of Safe Flight Area for UAV Inspection for Transmission Line Based on the Electromagnetic Field Calculation. Shandong Electr. Power.

[33] Peng Y., Ruan J.J. (2006). Calculation of Three-dimensional Harmonic Electric Field around Ultra High Voltage Overhead Line Based on the Charge Simulation Method. High Volt. Eng..

[34] McLaughlin R. (2006). Extracting Transmission Lines from Airborne LIDAR Data. IEEE Geosci. Remote. Sens. Lett..

[35] Wang X., Zhao J., Wu X., Zhang G., Pei C. (2011). Calculation method of space electric field in AC transmission line crossing area. High Volt. Technol..

[36] Huang F. (2008). Simulation Study on Environmental Impact of Overhead Line Electromagnetic Field. Master’s Thesis.

[37] Lee B., Park J., Myung S., Min S., Kim E. (1997). An effective modelling method to analyze the electric field around transmission lines and substations using a generalized finite line charge. IEEE Trans. Power Deliv..

[38] Yang W., Liu W., Zhao Q., Du Y. (2017). Detecting System Design of UAV Airborne Electromagnetic Field of Power Line Patrol. J. Chengdu Univ..

[39] Lin W., Cui Z.Z., Xu L.X., Xu Y.X. (2006). Signal Extraction of Electrostatic Target based on Array Detection. J. Proj. Rocket. Missiles Guid..

[40] Ning Y., Zhang W. (2019). Research on Locating Algorithm of Unmanned Airport Source Based on Array Detecting Line. J. Phys. Conf. Ser..

[41] Zhou M.C., Yan J., Liu J.W. (2004). Detection and Measurement.

